# Long-term Efficacy and Safety of a Hyaluronic Acid–Based Dermal Filler With Tri-Hyal Technology to Enhance Lip Volume

**DOI:** 10.1093/asjof/ojae110

**Published:** 2025-01-28

**Authors:** Philippe Kestemont, Ferial Fanian, Philippe Garcia, Anne Grand-Vincent, Laurent Benadiba, Henry Delmar, Isaac Bodokh, Patrick Brun, Frédéric Braccini, Christophe Desouches, Jérôme Paris, Ismahane Guimiot, Catherine Salomon, Patrick Trévidic

## Abstract

**Background:**

Interventions to enhance lip volume and shape are common aesthetic procedures to counter signs of aging.

**Objectives:**

This study aimed to assess the efficacy of Art Filler Lips (AFL), with or without retouching, at restoring lip shape and volume, tolerability, and persistence over 18 months.

**Methods:**

During this open-label study, AFL (≤2.0 mL) was injected into the upper, lower, and/or both red lips and borders at baseline (D0). Patients were evaluated at D21, when, if necessary, retouching was performed. Patients were evaluated at D42, D90, D180, D270, D360, D450, and D540. The primary assessment was based on evaluation at D21 using the Medicis Lip Fullness Scale (MLFS). Satisfactory volume restoration was defined as an improvement of ≥1 point vs D0. Secondary outcomes were adverse events (AEs), and investigator and patient satisfaction rates based on Global Aesthetic Improvement Scores.

**Results:**

Among 73 patients (97% females; 54.4 ± 10.5 years), 56% of lips were injected without any retouch. Mean MLFS scores either for upper and lower lips separately or together, significantly improved by D21 for the patients without retouching or D42 for those with (all *P* < .0001). At D21/42, 99% of upper lips, 94% of lower lips, and 96% of both lips showed satisfactory volume restoration. This proportion declined between D21/42 and D540. The most reported immediate AEs were swelling, sensitivity, and pain and were mild to moderate, lasting for <2 weeks.

**Conclusions:**

AFL is well tolerated and produces a sustained objective and subjective lip volume restoration and shape.

**Level of Evidence: 4 (Risk):**

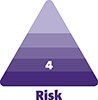

Interventions to enhance lip volume and shape are common aesthetic procedures to counter signs of aging and achieve an attractive shape.^1,2^ As females age, their lips become thinner and less well-defined, with philtrum enlargement and atrophy of the upper red lip, while older Caucasian males show similar reductions in hard and soft tissue volume, with concomitant thinning of the vermilion and cutaneous lip areas.^1-3^ Aesthetic procedures are undertaken to counter these signs of aging by recreating a youthful shape and ratio,^4^ and practitioners need to know the age-related changes and their influence on the optimal aesthetic outcomes for an individual patient.^1-3,5-8^

The authors of an American study to assess the most attractive lip dimensions of white females based on a survey reported that the majority of survey takers chose 1:1 as the most attractive ratio between the upper and lower lips.^9^ Therefore, when females undergo aesthetic procedures to enhance lip volume, the natural ratio of 1:1 should be preserved or achieved.^9^ Lip augmentation techniques include hyaluronic acid (HA) soft-tissue fillers, fat grafting, and soft alloplastic implants.^1,3,10-14^ HA fillers have largely replaced polyacrylamide, reflecting concerns that the latter may be associated with granuloma formation.^11^ Previous studies have shown that lip augmentation has a comparatively shorter duration relative to other facial areas, and treatments only remain effective in about half the treated patients after 12 months.^15,16^ The diversity of augmentation techniques and HA-based fillers on the market emphasizes the need for studies assessing the long-term efficacy of these products.

Art Filler Lips (AFL; Laboratoires FILLMED, Paris, France) is an injectable viscoelastic gel HA filler for improving the shape or increasing volume of the lips as well as correcting medium to deep skin depressions. It has Tri-Hyal technology, a proprietary combination of 3 types of crosslinked HA designed to optimize smoothing and volatizing results, and it contains 0.3% lidocaine hydrochloride to enhance patient comfort during the procedure. This open-label study evaluated the efficacy, safety, and persistence of AFL in lip augmentation, with or without reinjection (during the study), over 18 months.

## METHODS

### Study Design

**Figure 1.**
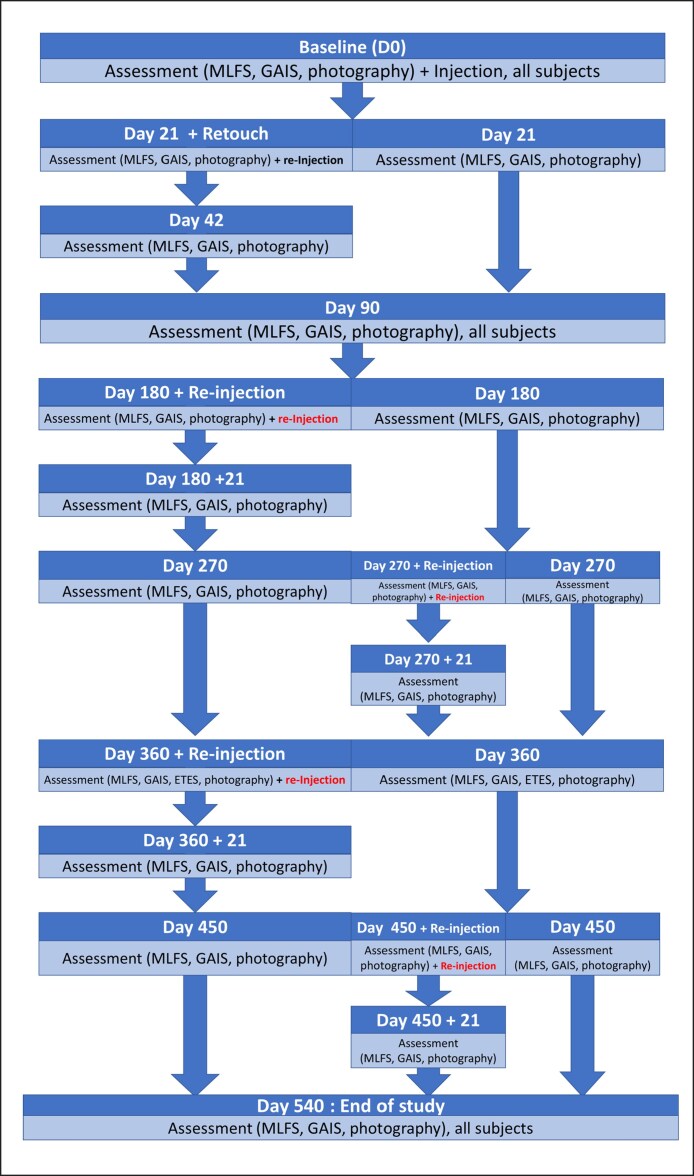
Study design. Assessment = Global Aesthetic Improvement Scale; Medicis Lip Fullness Scale.

This open-label long-term study was performed at 8 centers in France from 2016 to 2020 by various specialists, including 3 plastic surgeons, 1 aesthetic physician, 3 head and neck plastic surgeons, and 1 dermatologist. They assessed the efficacy and tolerability of AFL during an 18-month follow-up study (Figure 1).

### Participants and Inclusion and Noninclusion Criteria

The study enrolled males or females aged 19 years or older, with a Fitzpatrick phototype of I to IV. In order to avoid overcorrection or unnecessary treatment, patients’ lips had to be assessed as Grade 1 or 2 on the validated 5-point Medicis Lip Fullness Scale (MLFS), which ranges from 1 (very thin lips) to 5 (very full lips).^17^ Patients could not plan to undergo a facial cosmetic procedure and aesthetic injections, such as botulinum toxin or dermal filler during the study duration. Noninclusion criteria included a lifetime history of facial injections and/or implants that employed nonabsorbable fillers or a skin-retaining device on the face, such as mesh, gold wire, liquid silicone, or other particulate material. Patients who underwent medical treatment by laser, ultrasound, deep chemical peeling, or facial dermabrasion in the previous 3 months or who planned to undergo such treatment during the study were excluded. People with a history of hypertrophic, keloid or dyschromic scar, multiple severe allergies or anaphylactic shock, or hypersensitivity to any ingredient were also excluded.

### Treatment and Assessments

AFL was injected at baseline (Day 0, D0) with a maximum allowable volume of 2.0 mL into both red lips and/or borders. Volume and injection technique were performed at the injector's discretion, but they were predominantly injected in the lip outline and red line, and a fan technique was used for 1% to 3% of treatments. Patients were evaluated on D21 for a possible touch-up injection, using a maximum of 1 mL AFL. In this case, patients were reevaluated on D42. As this is an open study, assessments were undertaken by the injectors, and all patients were evaluated at D90 (3 months), D180 (6 months), D270 (9 months), D360 (12 months), D450 (15 months), and D540 (18 months). During the 18-month study, patients could receive a maximum of 2 reinjections of AFL with a minimum interval of 6 months at D180/D270 (only for patients who had not been injected at D180) and D360. If deemed necessary by the patient and clinician, a retouch could be performed, using a maximum of 1 mL AFL 3 weeks after each of these injections. Figure 1 summarizes the study design.

The primary assessment was the increase in volume of each treated area (upper, lower, and/or both lips) on D21 after the first injection; based on standard photographic visual scales, investigators rated the appearance on the 5-point MLFS.^17^ Satisfactory volume restoration was defined as an improvement of ≥1 on the MLFS, and the success rate was defined as the percentage of patients with ≥1 grade improvement on the MLFS evaluated at each time point. If a retouch was deemed necessary by the patient and clinician at D21, an additional assessment was completed on D42. The persistence of the correction was evaluated at every visit. Patients who underwent reinjection at any visit were also evaluated 3 weeks after the injection session. Local and general adverse events (AEs) were recorded at each study visit. Investigators and patients also assessed outcomes on the Global Aesthetic Improvement Scale (GAIS) at each visit.

### Ethical Approval

Ethical approval was provided by the ethical committee of Île-de-France VI (Comité de protection des personnes), Pitié-Salpêtrière Hospital, Paris (Trial registration: ID-RCB: 2016-A00358-43). All patients provided a written informed consent, by which the patients agreed to the use and analysis of data. The study was conducted in accordance with ISO 14155:2011.

### Statistical Analysis

Satisfactory volume restoration was defined as an improvement of ≥1 point on the MLFS compared with baseline assessed separately for the upper, lower, and both lips.^17^ A satisfactory volume correction rate of 95% was expected at D21 (D42 for the retouch group). To detect this rate with an accuracy of 5% (95% CI), 73 treated lips were required. Therefore, the study aimed to assess at least 100 lips.

The analyses encompassed 3 populations. The intention to treat (ITT) encompassed all patients enrolled. The per-protocol (PP) population included patients with results at D21 after the first injection or D42 in those who received a retouch. The safety population included all patients who received at least 1 AFL injection.

The MLFS analysis was performed on the ITT population based on the mean evolutions between baseline and each time point for the lower, upper, and both lips. The statistical significance of the change between baseline and D21 or D42 was assessed using the Wilcoxon test. Analyses were repeated using the PP population. Data from D21 if the patient was only injected on D0, or from D42 if the patient was reinjected on D21, were combined and analyzed together to also give an “optimized” D21 value.

GAIS scores were evaluated compared to baseline, including 7 grades from +3 for very strongly improved to −3 for very strongly worsened and were assessed at each time point for the lower, upper, and both lips. This analysis was performed in the PP population.

## RESULTS

### Baseline Characteristics and Injections

**Figure 2.**
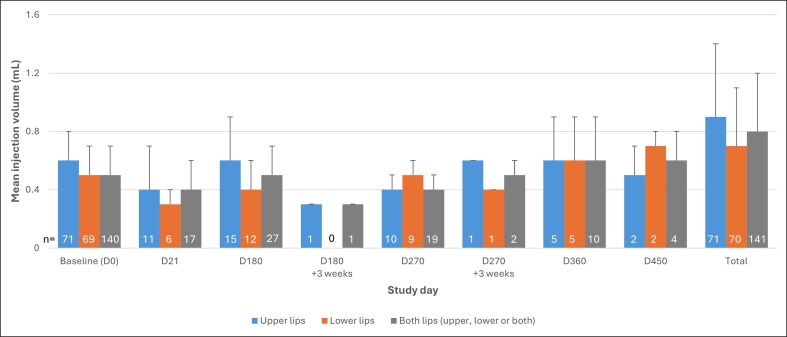
Mean volume (±standard deviation [SD]) of filler injected at each study visit (intention-to-treat population). Data from 2 patients were not included in the analysis of SD. Two patients were injected at baseline although their Medicis Lip Fullness Scale (MLFS) was 3 on D0 (inclusion criteria were MLFS 1 or 2); data regarding these patients were removed from the per-protocol analyses.

The study enrolled 73 healthy Caucasian patients (97% females), with a mean age of 54.4 ± 10.5 years (mean ± standard deviation [SD]). Table 1 summarizes the demographics and baseline MLFS scores. Figure 2 shows the volume injected over the course of the study: 56% of lips were injected once (without any touch-up), 33% of lips were reinjected once, 9% were reinjected twice, and 1% were reinjected 3 times. Not all patients opted to continue for the duration of the study: 171 lips were assessed at baseline, 133 lips were assessed at D360, and 42 lips at D540. The 27 G needles were used for most of the treatments (73%-100% of injections across all treatment sessions), followed by cannulas (up to 20% of injections across all treatment sessions). For 9% of injections, the data for injectors (needle or cannula) are missing.

**Table 1.** Demographics and Baseline MLFS Scores (Intention-to-Treat Population)

Characteristic		*n* = 73
Gender	Male, *n* (%)	2 (3)
	Female, *n* (%)	71 (97)
Age (years)	Mean (±SD)	54.4 (±10.5)^a^
	Median (range)	56 (21-76)
Fitzpatrick phototype	I, *n* (%)	10 (6)
	II, *n* (%)	32 (37)
	III, *n* (%)	25 (43)
	IV, *n* (%)	6 (14)
BMI (kg/m^2^)	Mean (±SD)	21.5 (±2.8)^a^
	Median (range)	21.4 (16.5-30.1)
Hormonal status (females only, *n* = 71)	Postmenopausal, *n* (%)	48 (66)
	Childbearing potential, *n* (%)	23 (32)
MLFS: upper lip (*n* = 71)	Grade 1 (very thin), *n* (%)	25 (34)
	Grade 2 (thin), *n* (%)	45 (62)
	Grade 3 (medium), *n* (%)	1 (1)^b^
	Grade 4 (full), *n* (%)	0 (0)
	Grade 5 (very full), *n* (%)	0 (0)
MLFS: lower lip (*n* = 70)	Grade 1 (very thin), *n* (%)	17 (23)
	Grade 2 (thin), *n* (%)	52 (71)
	Grade 3 (medium), *n* (%)	1 (1)^b^
	Grade 4 (full), *n* (%)	0 (0)
	Grade 5 (very full), *n* (%)	0 (0)
MLFS: both lips (*n* = 141)	Grade 1 (very thin), *n* (%)	42 (30)
	Grade 2 (thin), *n* (%)	97 (69)
	Grade 3 (medium), *n* (%)	2 (1)^b^
	Grade 4 (full), *n* (%)	0 (0)
	Grade 5 (very full), *n* (%)	0 (0)

MLFS, Medicis Lip Fullness Scale; SD, standard deviation. ^a^Data from 2 patients were not included in the analysis of SD. ^b^Two patients had been injected, although their MLFS was 3 on D0 (inclusion criteria were MLFS 1 or 2). Data regarding these patients were removed from the per-protocol analyses.

### Initial Efficacy

**Figure 3.**
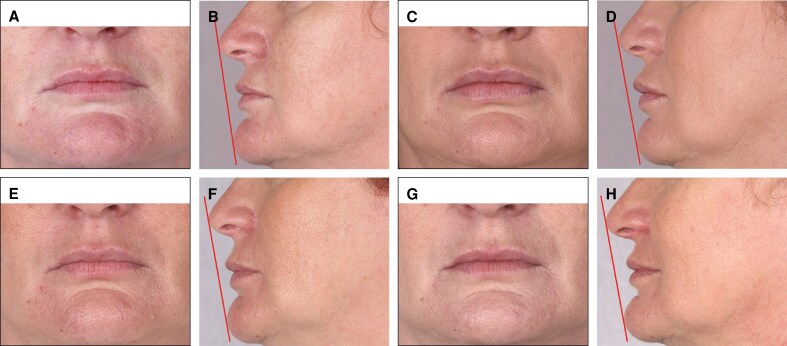
Photographs illustrating treatment in a 49-year-old female with baseline Medicis Lip Fullness Scale (MLFS) of 2 (A, B) before injection and (C, D) at D21, MLFS of 4, at D360, (E, F) MLFS of 3, and (G, H) at D540, MLFS of 2, after treatment with 0.6 mL Art Filler Lips in the upper lip and 0.6 mL in the lower lip on D0.

In the ITT population, the mean MLFS score for the upper lips increased from 1.7 ± 0.5 at baseline to 3.0 ± 0.6 at D21 for both nonretouched patients and for the combined optimized D21 (combines D21 data for nonretouched patients and D42 data for retouched patients; both *P* < .0001). For lower lips, the mean score increased from 1.8 ± 0.5 to 3.0 ± 0.7 between baseline and D21 for nonretouched patients and 3.1 ± 0.7 for optimized D21 (both *P* < .0001). The mean score for both lips increased from 1.7 ± 0.5 to 3.0 ± 0.7 between baseline and D21 and 3.1 ± 0.7 for D42, respectively (both *P* < .0001; Table 2, Figure 3). The results in the PP population (data not shown) were very similar to the ITT population.

**Table 2.** Medicis Lip Fullness Scale (Intention-to-Treat Population)

MLFS score	Upper lip (*n* = 71)			Lower lip (*n* = 70)			Both lips (*n* = 141)		
	D0	D21	Opt D21^a^	D0	D21	Opt D21^a^	D0	D21	Opt D21^a^
1: very thin, *n* (%)	25 (35)	1 (1)	1 (1)	17 (24)	0 (0)	0 (0)	42 (30)	1 (1)	1 (1)
2: thin, *n* (%)	45 (63)	12 (17)	10 (14)	52 (74)	14 (20)	12 (17)	97 (69%)	26 (19)	22 (16)
3: medium, *n* (%)	1 (1)^b^	45 (63)	45 (63)	1 (1)^b^	43 (61)	44 (63)	2 (1%)^b^	88 (62)	89 (63)
4: full, *n* (%)	0 (0)	13 (18)	15 (21)	0 (0)	11 (16)	12 (17)	0 (0)	24 (17)	27 (19)
5: very full, *n* (%)	0 (0)	0 (0)	0 (0)	0 (0)	2 (3)	2 (3)	0 (0)	2 (1)	2 (1)
Success,^c^ *n* (%)	—	68 (96)	70 (99)	—	64 (91)	66 (94)	—	132 (94)	136 (96)
Mean ± SD^d^	1.7 ± 0.5	3.0 ± 0.6	3.0 ± 0.6	1.8 ± 0.5	3.0 ± 0.7	3.1 ± 0.7	1.7 ± 0.5	3.0 ± 0.7	3.1 ± 0.7
Median (range)	2 (1-3)	3 (1-4)	3 (1-4)	2 (1-3)	3 (2-5)	3 (2-5)	2 (1-3)	3 (1-5)	3 (1-5)
*P*-value vs D0	—	<.001	<.001	—	<.001	<.001	—	<.001	<.001

SD, standard deviation. ^a^Optimized (Opt) D21 was calculated from values from D21 if the patient was only injected on D0 or from D42 if the patient was reinjected on D21. ^b^Two patients had been injected although their MLFS was 3 on D0 (inclusion criteria were MLFS 1 or 2). Data regarding these patients were removed from the per-protocol analyses. ^c^Success was defined as ≥1 grade improvement compared with DO. ^d^Data from 2 patients were not included in the analysis of SD.

**Figure 4.**
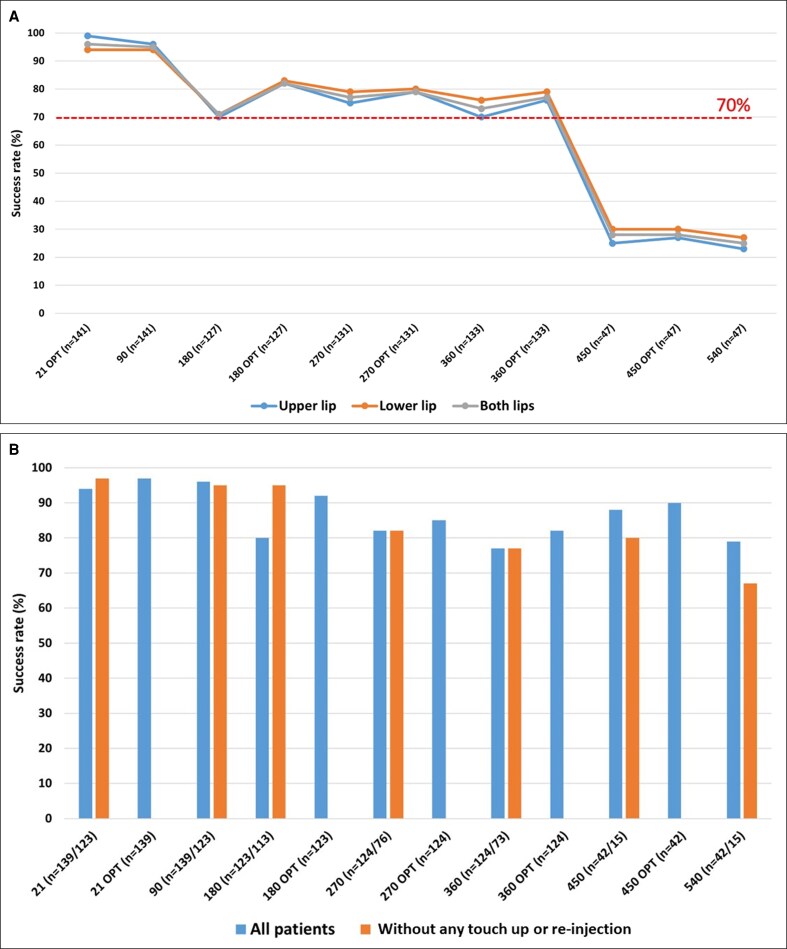
(A) The persistence of Medicis Lip Fullness Scale (MLFS) scores for both upper and lower lips (intention-to-treat population). *n*: number of injections in both or either lip; OPT: patients showing a satisfactory result ± retouching and reassessment after an additional 3 weeks; success rate: proportion of patients showing a satisfactory (≥1 grade) improvement. (B) Persistence of MLFS scores with and without touch-up or reinjection for both lips (per-protocol population). *n*: all patients/no touch-up/reinjection (injected only at D0); OPT: patients showing a satisfactory result ± touch-up and reassessment after an additional 3 weeks; success rate: proportion of patients showing a satisfactory (≥1 grade) improvement.

In the ITT population at D21, 96% of injections in the upper lip, 91% in the lower lip, and 94% in both lips showed ≥1 point improvement in MLFS scores (all *P* < .0001). Including those who received retouching, at D42, 99% of injections in the upper lip, 94% in the lower lip, and 96% in both lips showed at least 1 point improvement in MLFS scores (all *P* < .0001; see Figure 4A). At baseline, 1% of patients showed Grade 3 (medium fullness) MLFS for the upper, lower, and both lips which did not meet the inclusion criteria (MLFS of 1 or 2) and were excluded from the PP population. At D21 (or D42, after reinjection where necessary), 63% of upper, lower, and both lips reached Grade 3. At baseline, no patients showed Grade 4 (full) or Grade 5 (very full) MLFS at any target zone. Among the 17 patients retouched at D21, 21% of upper lips, 17% of lower lips, and 19% of both lips met the MLFS Grade 4 at D42. In addition, 3% of lower lips and 1% of both lips met the MLFS Grade 5 at this time point.

### Persistence

**Figure 5.**
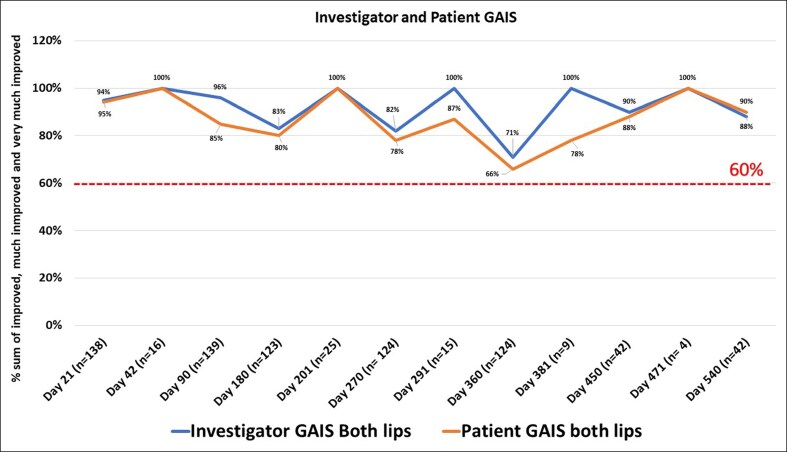
The percentage of investigator and patient Global Aesthetic Improvement Scale scores for both lips as the sum of improved, much improved, or very much improved (per-protocol population). *n*: number of injections in both or either lip; +3 weeks: patients who received a retouch were reassessed after an additional 3 weeks.

The success rate of volume restoration was 99% on D21 for upper lip, 96% for lower lip, and 97% for both lips. The evolution of the success rate over time is demonstrated in Figure 4A. This evolution varies between the patients who received an additional injection vs those who only had 1 injection on D0 (Figure 4B). For example, patients who were injected at D180 showed a drop off before this date and an increased success rate after reinjection, with a similar pattern around the other reinjection points, reflecting the longevity of the gel. The percentage of investigators that rated GAIS lip fullness as improved, much improved, or very much improved was >70% for all time points. The satisfaction rate was >65% for patients GAIS across the study (Figure 5).

### Safety and Tolerability

Table 3 summarizes the AEs. A total of 48 patients (46% of the total population) reported 80 AEs during the study, of which 9 were deemed not related to the injections (fever, allergic rhinitis, lung infection, dental infection, flu, bronchitis, herpes on buttocks, and diverticulitis). The AEs reported to be related to injection ranged from mild (*n* = 44, 62%) and moderate (*n* = 26, 37%) to severe intensity (*n* = 1, 1%, edema at D90). The most reported were swelling, sensitivity, and pain, and 85% lasted for <2 weeks. One patient discontinued due to AEs (breast cancer).

**Table 3.** Treatment-Related Adverse Events (Safety Population)

	Upper lip (*n* = 71)	Lower lip (*n* = 70)	Both (*n* = 73; 141 injections)
≥1 Treatment-emergent AE, *n* (%)	23 (32)	21 (30)	32 (45)
Nodule, *n*	18	12	31
Pain on palpation, *n*	9	6	15
Edema^a^, *n*	5	2	7
Overcorrection, *n*	2	3	5
Hematoma, *n*	3	1	4
Tyndall effect, *n*	1	2	3
Dyschromia, *n*	1	1	2
Induration, *n*	1	1	2
Ecchymosis, *n*	1	0	1
Spontaneous pain, *n*	1	0	1

AE, adverse event. ^a^All edema cases resolved within 3 weeks, except 1 at D90.

## DISCUSSION

This study adds to a growing body of evidence that HA fillers are effective and well tolerated when used for lip augmentation.^3,10-16^ The primary assessment was performed on D21 after the first injection for both safety and efficiency, which corresponds to the most common evaluation period followed in daily practice. The 3 monthly assessments allowed us to continue to evaluate the efficacy and safety, and discouraged withdrawal from the study. In the ITT population at optimized D21, which includes those who received retouching after the initial injection, 99% of AFL injections in the upper lip, 94% in the lower lip, and 96% in both lips showed at least 1 point improvement in MLFS scores (all *P* < .0001). Interestingly, at D90, in the PP population without touch-up or reinjection, 95% of AFL injections in both lips showed ≥1 point improvement in MLFS scores, which is superior to the lip fullness success rates obtained with Juvéderm Volbella with Lidocaine (Allergan, Santa Barbara, CA) and Restylane-L (Medicis Aesthetics, Scottsdale, AZ), which were 84% and 81%, respectively, 3 months after the last injection.^18^ At baseline, 1% of patients showed Grade 3 (medium) MLFS for the upper, lower, and both lips, which increased to 63% at optimized D21.

The proportion of patients who showed satisfactory volume restoration declined between D21 and D540, mirroring the results from a recent study and meta-analysis.^15,16^ Nevertheless, AFL showed a sustained objective (MLFS) and subjective (investigator and patient GAIS) benefit that persisted in some patients for up to 18 months. At D360, 77% of all studied patients, and the same proportion without any retouch, showed ≥1-point MLFS improvement. At D540, 79% of all the studied patients and 67% of patients without any retouching still showed a satisfactory result. The sustained improvement was consistent in both upper and/or lower lips and across multiple assessment methods, suggesting that the findings are robust and clinically meaningful, despite the reduced number of patients continuing the study to D540. The beneficial effects were persistent and remained significant 18 months postinjection and were consistent with the results obtained from a previous study.^19^ However, the number of patients in some of these subgroups was relatively small.

AFL was well tolerated during long-term follow-up with no serious events reported. Treatment-related AEs tended to be mild or moderate, transient, and probably related to the procedure rather than the filler, consistent with previous results.^16^ The injections by AFL were well tolerated by the patients and induced mainly some minor adverse effects, including slight pain during injection, erythema or edema, or pain just after injection. Most of them were resolved within 14 days after injection. These reported adverse effects were those typically expected following HA filler injection, confirming the safety of the tested filler for the treatment of lips in this study.^19-21^ Accurate anatomical placement and injecting the minimum volume of filler needed to obtain the required aesthetic outcome are important to avoid “sausage” or “duck” lips or a “trout pout” as well as to minimize AEs.^2-4^ The total mean volume injected during the study (0.9 ± 0.5 mL) was consistent with other studies assessing HA fillers for lip augmentation; the total volumes of filler required for treatment with other fillers were, on average, 1.54 to 1.82 mL Restylane Kysse with Lidocaine (Galderma, Upssala, Sweden), 1.82 to 1.97 mL Juvéderm Volbella with Lidocaine (Allergan), and 1.86 mL Restylane-L (Medicis Aesthetics).^13,14,22,23^

The study has certain limitations, including being nonrandomized and open label. However, it is not possible to do a split face study on lips, and grouped studies are very difficult to implement as the shape of the lips is very individualized. Furthermore, the study aimed to mirror practice in the real world. However, the consistent results across multiple assessment methods and anatomical sites suggest that the findings are robust and clinically meaningful. The study enrolled Caucasians, and the patients were predominately female; future studies could enroll more males and different ethnic cohorts, who have different optimal lip sizes and facial ratios.^2^ Given the different outcomes desired by young and older females, a study that is adequately powered to detect age-related differences might be insightful.^4^ Furthermore, the numbers of patients in each center were too small to discern any difference between sites or practitioners.

## CONCLUSIONS

AFL injections in the upper and/or lower lips showed at least 1 point improvement in MLFS scores in almost all patients. The proportion of patients who showed satisfactory volume restoration declined over 18 months from 99% to 71%. Nevertheless, AFL shows a sustained objective and subjective benefit that was consistent and persisted in some patients for at least 18 months, suggesting the findings are clinically meaningful. AFL was well tolerated, suggesting that this is a safe and effective product for lip augmentation.
